# Re-evaluating the necessity of routine laboratory tests after high tibial osteotomy surgery

**DOI:** 10.1186/s12891-021-04608-8

**Published:** 2021-08-23

**Authors:** Hong-bo Li, Si Nie, Min Lan, Xin-gen Liao, Zhi-ming Tang

**Affiliations:** 1 Department of Orthopedics, Jiangxi Provincial People’s Hospital Affiliated to Nanchang University, No. 92 Aiguo Road, Donghu District, Jiangxi Province 330006 Nanchang, People’s Republic of China; 2 Department of Radiology, Jiangxi Provincial People’s Hospital Affiliated to Nanchang University, 330006 Nanchang, People’s Republic of China

**Keywords:** High tibial osteotomy, Postoperative clinical interventions, Postoperative laboratory tests, Risk factors

## Abstract

**Background:**

To assess the utility of routine postoperative laboratory tests for patients undergoing high tibial osteotomy (HTO) surgery.

**Methods:**

The associations between clinical risk factors and postoperative clinical treatment were analyzed. Additionally, a logistic regression analysis was performed to detect independent risk factors for patients requiring postoperative clinical treatment.

**Results:**

A total of 482 patients with symptomatic isolated medial compartment osteoarthritis from January 2015 to May 2020 were included in the present study and underwent examination by the full set of postoperative laboratory tests within 3 days after HTO surgery. However, only a small proportion of the patients with anemia (3.9 %), hypoalbuminemia (4.1 %), and abnormal serum potassium levels (3.5 %) required clinical intervention after surgery. Binary logistic regression analysis showed that the body mass index (BMI), preoperative hemoglobin level, estimated blood loss and operative duration were independent risk factors for postoperative blood transfusion in patients who underwent HTO surgery, and factors associated with albumin supplementation were female sex and preoperative albumin level. In addition, these results indicated that preoperative potassium was potential risk factor for patients who required potassium supplementation postoperatively.

**Conclusions:**

Based on the analysis, we conclude that routinely ordering postoperative laboratory tests after HTO surgery is unnecessary. However, for patients with identified risk factors, routine postoperative laboratory tests are still needed.

## Background

Medial opening-wedge high tibial osteotomy (HTO) is an established treatment for medial compartment knee osteoarthritis with varus deformity [1]. As a joint preservation procedure, the surgery is designed to restore the mechanical axis of the lower extremity, transfer the load from the medial compartment to the nonaffected lateral compartment, and subsequently delay the progression of osteoarthritis [2].

Laboratory tests within 3 days after major orthopedic surgery are ordered to investigate many clinical details and potentially serious complications [3]. However, after the concept of clinical practice guidelines for enhanced recovery after surgery was gradually introduced and promoted to the field of HTO surgery, orthopedic surgeons were increasingly encouraged to minimize the use of invasive surgery techniques, improve perioperative blood management. In particular, the widespread use of tranexamic acid is a successful milestone in perioperative blood management and perioperative care pathways in the HTO surgery field [4, 5].

Recently, several studies have consistently revealed that routine postoperative laboratory tests are not necessary for patients following orthopedic surgery or even joint replacement surgery, unless patients have a history of preoperative abnormal laboratory test [6–8]. However, few studies have reported the necessity of routine laboratory tests in patients following HTO surgery.

Thus, this study aimed to re-evaluate the necessity of routine postoperative laboratory tests for patients following HTO surgery. In addition, we investigated the correlations between clinical parameters and abnormal postoperative laboratory test results and identified the risk factors associated with abnormal laboratory test required clinical interventions.

## Methods

### Patient selection

This retrospective study was approved by the medical research ethics committee of our hospital. The diagnosis of patients with OA primarily relies on clinical evidence, standing X-rays, and magnetic resonance imaging (MRI) examination. Operative therapy is indicated depending on the degenerative process and evaluated by the K-L classification on X-ray examination.

Patients undergoing HTO for other indications (cartilage resurfacing or ligament reconstruction) and patients with a previous tibial or femoral fracture were excluded from this study. Furthermore, patients presenting with a history of antiplatelet therapy, malignant tumors, significant hematological disorders, and infectious or systematic inflammatory disease were excluded from this study.

### Data collection

HTO was performed or directly supervised by a consultant surgeon. All patients’ demographics and clinical characteristics were carefully recorded, including age, sex, body mass index (BMI), comorbidities (diabetes mellitus, high blood pressure, alcohol consumption status and smoking status), as well as surgery-related factors (intraoperative blood loss, transfusion, operative duration and tranexamic acid use), preoperative and postoperative routine laboratory test results, and any medical intervention that was directly related to abnormal laboratory values (blood transfusion, albumin supplementation, and electrolyte supplementation).

The normal reference ranges for laboratory values at our institution are listed in Table 1. Aggressive interventions were not routinely performed for patients with abnormal preoperative laboratory tests before HTO surgery unless they met the criteria of clinical intervention. Patients with a hemoglobin level < 70 g/L or symptomatic anemia with a hemoglobin level of < 100 g/L, an albumin level < 30 g/L, and a serum potassium level < 3.5 mmol/L were considered to require clinical intervention [9]. During the study period, all HTO surgery patients routinely received an intravenous injection of 1.5 g of tranexamic acid 30 min before the incision and an articular injection of 0.5 g of tranexamic acid after the incision was sutured.

**Table 1:** The normal reference ranges for laboratory values

Laboratory Tests	Reference range
Routine blood test	
Hemoglobin (g/L)	115–150
Platelets (*10^9/L)	125–350
Liver function test	
Alanine aminotransferase (IU/L)	9–50
Aspartate aminotransferase (IU/L)	15–40
Albumin (g/L)	40–55
Renal function test	
Creatinine (umol/L)	44–115
Blood urea nitrogen (mmol/L)	1.70–8.30
Coagulation function	
PT (s)	10–14
APTT (s)	25–32
Electrolytes	
Serum sodium (mmol/L)	137–147
Serum potassium serum potassium (mmol/L)	3.5–5.3
Serum calcium (mmol/L)	2.0–2.6

*PT* prothrombin time, *APTT* activated partial thromboplastin

### Surgical techniques

An identical operative approach was applied in all patients. The arthroscopic evaluation and treatment of additional intraarticular lesions were performed after the application of a tourniquet. An approximately 8-cm-long skin incision was made along the anteromedial portion of the proximal tibia, and the medial collateral ligament was elevated from the subperiosteal bone. Then, two K-wires were threaded to the medial tibial diaphysis toward the fibular head under fluoroscopic guidance. The proximal tibia was cut on the medial, anterior, and posterior cortices under the guide wire, leaving the lateral cortex intact with the use of an oscillating saw. The mechanical axis was then adjusted according to the preoperative plan, and the correction was retained with a bone spreader. Then, the HTO plate was inserted into a subcutaneous tunnel and centered on the anteromedial plane of the tibia. Patients were allowed weight bearing as tolerated after the drain was removed. Patients were able to advance their weight bearing as pain and strength allowed with two crutches for 6 weeks, and full weight-bearing was permitted 6 weeks after surgery.

### Statistical analysis

Qualitative variables are expressed as frequencies and percentages and were assessed by the chi-square test. Quantitative variables are presented as the mean ± standard deviation, and the two groups were compared using independent-samples t tests. A binary logistic regression model was used to identify the independent risk factors for patients with abnormal laboratory test results who required postoperative clinical treatment. The receiver operating characteristic (ROC) curve was used to assess the predictive value of risk factors for postoperative clinical treatment. All analyses were performed using SPSS version 22 (SPSS, Inc., Chicago, IL). *P* < 0.05 was considered statistically significant.

## Results

### Laboratory test characteristics

Abnormal postoperative laboratory test results were recorded. Finally, the incidence and risk factors for patients requiring postoperative clinical treatment were analyzed. Hypoalbuminemia accounted for most abnormal postoperative laboratory test results, occurring in 44.0 % (212 cases) of the patients, followed by postoperative aspartate aminotransferase (36.9 %) and hemoglobin (22.0 %) abnormalities. However, only a small proportion of the patients received medical intervention that is directly related to the anemia (3.9 %), hypoalbuminemia (4.1 %), and abnormal serum potassium levels (3.5 %) (Table 2).

**Table 2:** Results of Routine Laboratory Tests for Patients Undergoing HTO Surgery

Laboratory Test (*n* = 482)	ALTR (n; %)	PCTR (n; %)
Routine blood test		
Hemoglobin	106(22.0 %)	19(3.9 %)
Platelets	36(7.5 %)	0 (0)
Liver function test		
Alanine aminotransferase	6(1.2 %)	0 (0)
Aspartate aminotransferase	178(36.9 %)	
Albumin	212(44.0 %)	20(4.1 %)
Renal function test		
Creatinine	77(16.0 %)	0 (0)
Blood urea nitrogen	2(0.4 %)	0 (0)
Coagulation function		
PT	30(6.2 %)	0 (0)
APTT	50(10.4 %)	0 (0)
Electrolytes		
Serum sodium	32(6.6 %)	0 (0)
Serum potassium	17(3.5 %)	17(3.5 %)
Serum calcium	19(3.9 %))	0 (0)

*ALTR* abnormal postoperative laboratory test result, *PCTR* postoperative clinical treatment required, *PT* prothrombin time, *APTT* activated partial thromboplastin time, *HTO* high tibial osteotomy

### Risk factors for patients requiring postoperative clinical treatment

#### Risk factors for patients requiring postoperative blood transfusion

Based on the analysis, there were no statistically significant differences in age or comorbidities between HTO patients with and without a postoperative blood transfusion (*P* > 0.05). However, the BMI, estimated blood loss and operative duration were significantly higher in patients with a postoperative blood transfusion than in those without a postoperative blood transfusion (*p* < 0.001). The preoperative hemoglobin level was significantly lower in patients with than without a postoperative blood transfusion (*p* < 0.001). Furthermore, females were more likely to require a blood transfusion (Table 3). Binary logistic regression analysis indicated that the BMI (OR = 0.584, *P* = 0.004), preoperative hemoglobin level (OR = 1.205, *P* < 0.001), estimated blood loss (OR = 0.991, *P* < 0.001) and operative duration (OR = 0.976, *P* = 0.024) were independent risk factors for postoperative blood transfusion in patients who underwent HTO surgery (Table 4).

**Table 3:** Postoperative Blood Transfusion for Patients with Abnormal Hemoglobin after HTO Surgery

Factor	Treatment Group (*n* = 19)	No Treatment Group (*n* = 463)	*P* value
Age (years)	53.58 ± 5.99	53.30 ± 5.92	0.846
Sex (n)			0.032
Male	8	317	
Female	11	146	
BMI (kg/m2)	26.25 ± 3.35	23.04 ± 2.88	*p* < 0.001
Smoking: n(%)	3(15.8 %)	133(28.7 %)	0.219
Alcohol use: n(%)	2(10.5 %)	94(20.3 %)	0.142
Diabetes mellitus: n(%)	31(5.8 %)	30(6.5 %)	0.390
High blood pressure: n(%)	4(21.1 %)	61(13.2 %)	0.306
Preoperative hb level (g/L)	104.05 ± 14.33	135.94 ± 12.20	*p* < 0.001
Estimated blood loss (mL)	489.47 ± 216.40	233.05 ± 87.34	*p* < 0.001
Operative time (minutes)	174.62 ± 47.62	145.74 ± 23.25	*p* < 0.001

*BMI* body mass index, *HTO* high tibial osteotomy

**Table 4:** Risk Factors for Postoperative Blood Transfusion in Patients Undergoing HTO Surgery

Risk factor	odds ratio	95 % confidence interval	*P* value
Sex	2.819	0.339–23.453	0.338
BMI	0.584	0.404–0.845	0.004
Preoperative hb level	1.205	1.096–1.325	*p* < 0.001
Estimated blood loss	0.991	0.986–0.996	*p* < 0.001
Operative time	0.976	0.955–0.997	0.024

*BMI* body mass index, *HTO* high tibial osteotomy

#### Risk factors for patients requiring postoperative albumin supplementation

The univariate and binary logistic regression analysis results showed that the female sex (OR = 0.163, *P* = 0.001) and preoperative albumin level (OR = 1.453, *P* < 0.001) were independent risk factors for patients who required albumin supplementation after HTO surgery (Tables 5 and 6).

**Table 5:** Clinical Characteristics of Patients Who Required Postoperative Albumin Supplement

Factor	Treatment Group (*n* = 20)	No Treatment Group (*n* = 462)	*P* value
Age (years)	54.65 ± 6.71	53.26 ± 5.88	0.303
Sex (n)			0.032
Male	7	318	
Female	13	144	
BMI (kg/m2)	23.87 ± 3.99	23.14 ± 2.92	0.283
Smoking: n(%)	2(10.0 %)	134(29.0 %)	0.064
Alcohol use: n(%)	2(10.0 %)	94(20.3 %)	0.392
Diabetes mellitus: n(%)	3(15.0 %)	30(6.5 %)	0.150
High blood pressure: n(%)	3(15.0 %)	63(13.6 %)	0.746
Preoperative Albumin (g/L)	34.99 ± 4.02	42.27 ± 5.22	*p* < 0.001
Estimated blood loss (mL)	285.00 ± 190.64	241.34 ± 102.47	0.076
Operative time (minutes)	141.25 ± 16.86	147.12 ± 25.50	0.308

*BMI* body mass index

**Table 6:** Risk Factors for Patients Requiring Postoperative Albumin Supplementation

Risk factor	odds ratio	95 % confidence interval	*P* value
Sex	0.163	0.057–0.470	0.001
Preoperative Albumin level	1.453	1.268–1.664	*p* < 0.001

#### Risk factors for patients requiring postoperative potassium supplementation

A significant difference was found between patients who did and did not require postoperative potassium supplementation in the preoperative potassium level and operative duration (*P* = 0.006 and *P* = 0.002; Table 7). Furthermore, binary logistic regression analysis was carried out to identify the independent risk factors for potassium supplementation in patients who underwent HTO surgery. The results showed that preoperative potassium (OR = 19.870, *P* < 0.001) was an independent risk factor for patients who required potassium supplementation postoperatively (Table 8).

**Table 7:** Clinical Characteristics of Patients Who Required Postoperative Potassium Supplementation

Factor	Treatment Group (*n* = 17)	No Treatment Group (*n* = 465)	*P* value
Age (years)	54.00 ± 6.86	53.29 ± 5.89	0.627
Sex (n)			0.159
Male	7	318	
Female	10	147	
BMI (kg/m2)	23.30 ± 3.74	23.16 ± 2.94	0.852
Smoking: n(%)	1(5.9 %)	135(29.0 %)	0.051
Alcohol use: n(%)	1(5.9 %)	95(20.4 %)	0.216
Diabetes mellitus: n(%)	2(11.8 %)	31(6.7 %)	0.327
High blood pressure: n(%)	3(17.6 %)	63(13.5 %)	0.716
Preoperative Potassium (g/L)	3.68 ± 0.53	4.10 ± 0.38	0.006
Estimated blood loss (mL)	264.71 ± 158.87	242.37 ± 105.41	0.401
Operative time (minutes)	136.59 ± 11.24	147.25 ± 25.51	0.002

*BMI* body mass index

**Table 8:** Risk Factor for Patients Requiring Postoperative Potassium Supplementation

Risk factor	odds ratio	95 % confidence interval	*P* value
Preoperative Potassium	19.870	4.805–82.166	*p* < 0.001
Operative time	1.025	0.997–1.054	0.082

### Diagnostic accuracy of risk factors for predicting postoperative clinical treatment

ROC curve analysis was performed to determine the value of risk factors for predicting postoperative clinical treatment in patients who underwent HTO surgery. The accuracy was analyzed by the area under the ROC curve (AUC). The results are demonstrated in Table 9. The results indicate that the preoperative hemoglobin level had the highest diagnostic accuracy for predicting transfusion in patients who underwent HTO surgery (AUC = 0.947, *P* < 0.001).

**Table 9:** Cutoff Values of Risk Factors for Patients Requiring Postoperative Clinical Treatment

Treatment	Risk factors	Cut-off value	Sensitivity	Specificity	AUC	*p*
Transfusion	BMI	23.15	89.5%	55.3%	0.770	<0.001
	Preoperative hb level	112.5	78.9%	97.0 %	0.947	<0.001
	Estimated blood loss	225	89.5%	81.2 %	0.888	<0.001
	Operative time	164.5	63.2%	81.4%	0.669	0.003
Albumin	Female gender	-	-	-	0.669	0.010
	Preoperative albumin level	38.95	95.0%	76.4 %	0.879	<0.001
Potassium	Preoperative potassium	3.45	41.2%	97.4%	0.707	0.004

*BMI* body mass index, *AUC* area under the curve

## Discussion

Recent years have witnessed unprecedented improvements in both modern-day surgical techniques and perioperative care pathways. In particular, the widespread use of tranexamic acid is a successful milestone of perioperative blood management and perioperative care pathways for HTO [9]. Recently, several studies have consistently revealed that routine postoperative laboratory tests are not necessary for patients after major orthopedic surgery. Instead, only patients with risk factors should undergo routine postoperative laboratory tests [9, 10]. To improve the quality of medical care and reduce medical expenses, many studies have recommended reducing the use of duplicative or limited clinical value laboratory tests [10].

Several studies have indicated that the more laboratory tests are performed on patients, the more likely they are to find something unusual [11]. Recently, several studies have consistently revealed that routine postoperative laboratory tests are not necessary for patients after major orthopedic surgery. Additionally, most of the information provided by these abnormal test results have no clinical value, and only patients with risk factors should undergo routine postoperative laboratory tests. Wu et al. [9] showed that up to 88.4 % of patients had abnormal postoperative laboratory results, and only 6.8 % of those patients required a postoperative medical intervention, indicating that routine postoperative laboratory tests are unnecessary for most patients. Serum laboratory tests are routinely ordered after HTO, but at our institution, the results are rarely abnormal. Furthermore, these infrequently abnormal serum laboratory values rarely require active intervention, which indicates that routine postoperative laboratory tests are not necessary for most patients after HTO surgery.

Park et al. [12] found that risk factors for transfusion include higher BMI, higher estimated blood loss, and lower preoperative hemoglobin levels. Wu et al. [9] retrospectively reviewed 349 patients who underwent primary elective unilateral total hip arthroplasty from January 2016 to November 2018 at a single institution, and a long operative duration and low preoperative hemoglobin level were the most predictive factors. Halawi et al. [13] found that BMI > 35 kg/m2, anemia and estimated blood loss > 250 mL were independent risk factors for transfusion. Greco et al. [14] have proposed that routine postoperative laboratory tests should be considered in patients presenting with a preoperative hemoglobin level below 130 g/l and postoperative clinical symptoms or signs of significant blood loss. Similarly, we also identified BMI > 23.15 kg/m2, preoperative hemoglobin < 112.5 g/L, estimated blood loss > 225 ml and operative duration > 164.5 min as independent risk factors for the requirement for a blood transfusion after HTO surgery.

In a retrospective study, Lin et al. [10] investigated 1915 patients who underwent lumbar spinal surgery and found that the incidence of abnormal postoperative serum albumin was 77.36 %, but only 1.95 % of those patients required albumin supplementation. In our study, the incidence of abnormal postoperative serum albumin as high as 44.0 % in patients after HTO, and we found that the incidence of albumin supplementation was 4.1 %, slightly greater than that reported by Lin et al. [10]. Hart et al. [15] reported that the female sex was an independent risk factor associated with the need for clinical treatment, possibly because female patients are always more likely to have anemia and hypoproteinemia. Wu et al. [9] showed that the female sex, a long operation, and a low preoperative albumin level were risk factors for patients requiring albumin supplementation. In accordance with previous studies, we found that risk factors for albumin supplementation were the female sex and a low preoperative albumin level.

In a retrospective study, Kildow et al. [7] investigated the results of basic metabolic panels of 767 patients and found that the incidence of potassium abnormalities was 25.2 % after primary total joint arthroplasty. Among the most significant risk factors for electrolyte abnormalities were the female sex, an advanced age, and diabetes. Lin et al. [10] reported that the incidence of abnormal electrolytes was low and that the predictive value was small; 0.92 % of the patients who underwent lumbar spinal surgery were diagnosed with hypokalemia postoperatively and required postoperative clinical treatment. Likewise, Greco et al. [14] demonstrated an incidence as great as 15.5 % of electrolyte supplementation in patients who underwent total joint arthroplasty. Most of these patients presented with a potassium level less than 4 mmol/l on preoperative testing. In line with previous studies, we found that 3.55 % of patients required potassium supplementation after HTO surgery. The preoperative potassium level was lower in patients who received potassium supplementation and was identified as an independent risk factor for postoperative clinical treatment in patients who underwent HTO surgery. The cutoff value was 3.45 mmol/L. This result suggests that a preoperative potassium level < 3.45 mmol/L is predictive of a high risk of postoperative clinical treatment.

To the best of our knowledge, this is the first study to evaluate the necessity of postoperative laboratory tests for Chinese patients after primary HTO. Although the results are interesting, there are still limitations to this study. First, this study was performed at a single tertiary academic center, and the relatively small sample may result in bias in the outcomes. Second, our transfusion criteria were somewhat subjective, as our protocol set a restrictive transfusion strategy according to a hemoglobin level < 70 g/L or symptomatic anemia with a hemoglobin level < 100 g/L, which might limit the generalizability of the study findings. Thus, further multicenter research with a larger sample is necessary to verify the efficacy of these risk factors in predicting postoperative medical intervention in patients following HTO surgery.

## Conclusions

In summary, based on the analysis, only a small number of patients required postoperative clinical interventions in response to abnormal laboratory values, and we conclude that routinely ordering postoperative laboratory studies after HTO surgery is unnecessary. However, for patients with identified risk factors, routine postoperative laboratory tests are still needed. We recommend selectively considering a postoperative complete blood count in patients with a BMI > 23.15 kg/m2, preoperative hemoglobin level < 112.5 g/l, estimated blood loss > 225 ml and operative duration > 164.5 min. Furthermore, we suggest selectively considering a postoperative basic metabolic panel in female patients with a preoperative potassium level < 3.45 mmol/l and preoperative albumin level < 38.95 g/L. However, multicenter studies with larger samples are necessary to verify our results.

## Data Availability

All the data will be available upon reasonable request to the corresponding author of the present paper.

## Abbreviations

HTO: High tibial osteotomy
BMI: Body mass index
MRI: Magnetic resonance imaging
K-L: Kellgren-Lawrence
ROC: Receiver operating characteristic
AUC: Under the curve
